# Risk factors for heart, cerebrovascular, and kidney diseases: evaluation of potential side effects of medications to control hypertension, hyperglycemia, and hypercholesterolemia

**DOI:** 10.3389/fcvm.2023.1103250

**Published:** 2023-06-02

**Authors:** Kazumitsu Nawata

**Affiliations:** Hitotsubashi Institute for Advanced Study (HISA), Hitotsubashi University, Kunitachi, Japan

**Keywords:** heart disease, cerebrovascular disease, kidney disease, side effects of medications, antihypertensive medications

## Abstract

**Background:**

Heart disease (HD), cerebrovascular disease (CBD), and kidney disease (KD) are serious diseases worldwide. These diseases constitute the leading causes of death worldwide and are costly to treat. An analysis of risk factors is necessary to prevent these diseases.

**Data and Methods:**

Risk factors were analyzed using data from 2,837,334, 2,864,874, and 2,870,262 medical checkups obtained from the JMDC Claims Database. The side effects of medications used to control hypertension (antihypertensive medications), hyperglycemia (antihyperglycemic medications), and hypercholesterolemia (cholesterol medications), including their interactions, were also evaluated. Logit models were used to calculate the odds ratios and confidence intervals. The sample period was from January 2005 to September 2019.

**Results:**

Age and history of diseases were found to be very important factors, and the risk of having diseases could be almost doubled. Urine protein levels and recent large weight changes were also important factors for all three diseases and made the risks 10%–30% higher, except for KD. For KD, the risk was more than double for individuals with high urine protein levels. Negative side effects were observed with antihypertensive, antihyperglycemic, and cholesterol medications. In particular, when antihypertensive medications were used, the risks were almost doubled for HD and CBD. The risk would be triple for KD when individuals were taking antihypertensive medications. If they did not take antihypertensive medications and took other medications, these values were lower (20%–40% for HD, 50%–70% for CBD, and 60%–90% for KD). The interactions between the different types of medications were not very large. When antihypertensive and cholesterol medications were used simultaneously, the risk increased significantly in cases of HD and KD.

**Conclusion:**

It is very important for individuals with risk factors to improve their physical condition for the prevention of these diseases. Taking antihypertensive, antihyperglycemic, and cholesterol medications, especially antihypertensive medications, may be serious risk factors. Special care and additional studies are necessary to prescribe these medications, particularly antihypertensive medications.

**Limitations:**

No experimental interventions were performed. As the dataset was comprised of the results of health checkups of workers in Japan, individuals aged 76 and above were not included. Since the dataset only contained information obtained in Japan and the Japanese are ethnically homogeneous, potential ethnic effects on the diseases were not evaluated.

## 1. Introduction

Heart disease (HD), cerebrovascular disease (CBD), and kidney disease (KD) are serious diseases. Ischemic HD (IHD), stroke (a type of CBD), and KD were the leading, second-leading, and tenth-leading causes of deaths globally in 2019, respectively (1). The World Health Organization (WHO) estimated (1) that IHD caused 8.9 million or 16% of the world's total deaths, stroke caused 6.2 million deaths, approximately 11% of the total deaths, and the deaths caused by KD totaled 1.3 million in 2019.

In the United States, HD, CBD (stroke), and KD were the leading, fifth leading, and tenth-leading causes of death in 2020. They caused 696,962 (20.6%), 160,264 (4.7%), and 52,547 (1.6%) deaths, respectively (percentages of total deaths are listed in parentheses) (2).

In Japan, HD, CBD, and KD were the second-, fourth-, and eighth-leading causes of death in 2020 (3). They caused 205,596 (15.0%), 102,978 (7.5%), and 26,948 (2.0%) deaths in 2020, respectively (percentages of total deaths are listed in parentheses). The medical expenditures in fiscal year 2019 (4) were 2.09 trillion yen for HD, 1.83 trillion yen for CBD, and 1.66 trillion yen for KD, respectively. These medical expenditures accounted for 12.6% of the total Japanese national medical expenditure of 44.39 trillion yen.

The American Heart Association (AHA) (5) described the risk factors for coronary HD. The major risk factors are classified into three categories: (i) non-modifiable risk factors that cannot be changed; (ii) modifiable risk factors that can be modified, treated, or controlled; and (iii) other factors that contribute to HD risks. Non-modifiable risk factors include age, sex, and heredity (including race). Modifiable risk factors include tobacco smoking, high blood cholesterol, total cholesterol, triglycerides, high blood pressure or hypertension, physical inactivity, obesity, being overweight, and diabetes. Other factors included stress, alcohol consumption, diet, and nutrition. Centers for Disease Control and Prevention (CDC) (6) described that the risk factors for HD and stroke are high blood pressure, low-density lipoprotein (LDL) cholesterol, diabetes, smoking and secondhand smoke exposure, obesity, unhealthy diet, and physical inactivity.

High blood pressure (BP) or hypertension is considered a major risk factor for HD or cardiovascular disease (CVD). Fuchs and Whelton (7) noted that hypertension had the strongest evidence of causation among the risk factors for CVD. The American College of Cardiology (ACC), AHA, and other organizations (8) presented a new guideline (the 2017ACC/AHA guideline) for hypertension in 2017. Under the new guideline, the criteria for hypertension lowered to 130/80 mmHg (SBP ≥ 130 or DBP ≥ 80 mmHg) from 140/90 mmHg (SBP ≥_ _140 or DBP ≥ 90 mmHg) of the JNC7 guideline settled in 2003 (9). Nawata (10) reported that 14.0% and 38.0% were classified as having hypertension according to the 140/90 and 130/90 mmHg criteria, respectively. Muntner et al. (11) mentioned that the 2017 ACC/AHA guideline would increase medication use and reduce the prevalence of CVD.

Many studies have analyzed the relationship between high BP or hypertension [especially high systolic BP (SBP)] and HD or CVD (12–23). Although most of these studies indicated that hypertension is a risk factor for HD and CVD, some questioned the relationship between them (13, 18–20, 22, 23).

Risk factors for CBD (stroke) are classified as non-modifiable and modifiable factors. The American Stroke Association (ASA) (24) stated that nonmodifiable risk factors include age, family history, race, gender, prior stroke history, transient ischemic attack (TIA), and heart attack. Modifiable risk factors include high BP, smoking, diabetes, diet, physical inactivity, obesity, high blood cholesterol, artery disease, peripheral artery disease, atrial fibrillation, and sickle cell disease. Various studies have been conducted on CBD (25–31). In case of CBD, long-term rehabilitation frequently becomes a serious problem in addition to prevention, detections, and treatment (25, 26, 30).

Furthermore, CDC (32) described diabetes and high BP as two important risk factors for chronic KD (CKD). HD, obesity, family history of CKD, inherited kidney disorders, past kidney damage, and older age were other risk factors. The American Kidney Fund (33) mentioned that diabetes is a leading risk factor, and high BP and race or ethnicity are other important risk factors. The Kidney Foundation of Canada (34) also mentioned that diabetes, high BP, and a family history of KD are risk factors. Since KD, especially CKD, is a serious global burden (35), many studies on its risk factors (36–40) and treatment procedures have been conducted (41–46). CKD is a costly disease to treat. Liyanage et al. (42) reported that “renal replacement therapy (RRT), through either dialysis or renal transplantation, is a lifesaving yet high-cost treatment for people with end-stage kidney disease.” Although the results of cost-effectiveness analyses (47–51) suggest that renal transplantation is more efficient than dialysis, few renal transplantations have been performed in Japan. The number of kidney transplantations in the United States (52) was 23,642 in 2020. In contrast, the numbers in Japan (53) were 216 and 127 in the fiscal years 2019 and 2020, respectively, and the number of dialysis patients (54) was 347,641 at the end of 2020. Nawata and Kimura (55) reported that the medical cost of an individual with KD was 14.5 times higher than that of an individual without KD.

In the present study, the risk factors for HD, CBD, and KD were analyzed using the JMDC Claims Database (56), which includes 13,157,681 medical checkups performed on 3,233,271 individuals in Japan. The side effects of medications to control hypertension (antihypertensive medications), hyperglycemia (antihyperglycemic medications), and hypercholesterolemia (cholesterol medications) on these diseases were also evaluated.

## 2. Data, design of the study and models

### 2.1. Data

In Japan, most employees aged 40 years or older undergo mandatory medical check-ups at least once a year under the Industrial Safety and Health Act. Younger employees and their family members may voluntarily undergo medical checkups. The JMDC Claims Database is a nationwide health information database that collects data from various health insurance societies and includes 13,157,681 medical check-ups obtained from 3,233,271 individuals from January 2005 to September 2019. The database contains various health information, including HD, CBD, and KD histories.

### 2.2. Design of the study and models

In this study, not only conventional risk factors but also the effects of antihypertensive, antihyperglycemic, and cholesterol medications were analyzed using logit (logistic regression) models.

In the rest of the paper, taking antihypertensive medications is referred to as “with antihypertensive medications” and not taking medications is referred to as “without antihypertensive medications.” This phrasing is similarly used for the other medications. Table 1 shows the percentages of observations with histories of HD, CBD, and KD classified as with and without medications. The percentages having disease histories of individuals with medications are several times larger than those without medications, as shown in “Ratio (a/b)” and “95% CI” [95% confidence intervals (CIs) are calculated using the formula given in Appendix A]. For HD history, the ratio of the percentages is 6.88 with a 95% CI of 6.82–6.94 between individuals with and without BP medications.

**Table 1:** Percentages of observations having HD, CBD, and HD histories by taking medications or not.

Disease	Mean (a)	SD	Obs.	Mean (b)	SD	Obs.	Ratio (a/b)	95% CI
	With antihypertensive medications			Without antihypertensive medications				
HD	7.37%	26.13%	11,80,666	1.07%	10.30%	93,07,991	6.88	6.82–6.94
CBD	3.91%	19.39%	11,80,246	0.45%	6.66%	93,08,321	8.78	8.67–8.90
KD	1.29%	11.28%	11,55,359	0.14%	3.78%	91,93,796	9.00	8.79–9.21
	With antihyperglycemic medications			Without antihyperglycemic medications				
HD	7.45%	26.25%	348,781	1.59%	12.49%	10,13,5861	4.70	4.64–4.76
CBD	3.23%	17.68%	348,628	0.75%	8.64%	10,13,5931	4.30	4.21–4.38
KD	1.34%	11.49%	341,206	0.23%	4.84%	10,00,3946	5.70	5.52–5.88
	With cholesterol medications			Without cholesterol medications				
HD	8.11%	27.30%	794,335	1.26%	11.16%	96,90,936	6.42	6.36–6.48
CBD	3.52%	18.42%	793,993	0.62%	7.83%	96,91,190	5.70	5.64–5.76
KD	1.01%	10.00%	783,142	0.21%	4.59%	95,62,642	4.79	4.73–4.85

SD, standard deviation; Obs., no. of observations; HD, heart disease; CBD, cerebrovascular disease; KD, kidney disease; 95% CI, 95% confidence interval calculated from the formulas in Appendix A.

However, we cannot say that taking medications increases the probability of developing diseases from these facts. If an individual once had these diseases, the medications would be continuously prescribed for treatment and recurrence prevention purposes afterward. Therefore, causality problems (i.e., diseases are causes, and prescriptions of medications are results) must be considered in the analysis. The following methods were used to avoid causality problems.

For the analysis of HD, individuals who had HD history data at both years t and t + 1, no HD history at year t (“without HD history” hereafter), and had data (either positive or negative) concerning HD at year t + 1 (i.e., the following year) were selected, and the same selection methods were used for CBD and KD. Logit (logistic regression) models were used for the analysis.

Define:

$HD_{t}$ (dummy variable) is 1 if an individual has a HD history at year t and 0 otherwise,
$CBD_{t}$ (dummy variable) is 1 if an individual has a CBD history at year t and 0 otherwise, and
$KD_{t}$ (dummy variable) is 1 if an individual has a KD history at year t and 0 otherwise.

We use the following three models in which $HD_{it+1}$, $CD_{it+1}$ and $KD_{it+1\;}$ are the dependent variables.

The basic design of the model is the models given by

(1) $$P[y_{t+1}|y_{t=0}]\;=\;\Lambda (x_{t}^{\prime }\beta )$$

where $y_{t}$ is a variable representing a disease history, Λ is the distribution function of the logistic distribution given by $\Lambda (\omega )\;=\;\frac{\exp(\omega )}{1\;+\;\exp(\omega )}$, and $x_{t}$ is a vector of covariates at t. It may be possible to extend the model to $\Lambda \left(\overset{k}{\underset{s\;=\;0}{\sum }}x_{it-s}^{\prime }\beta_{s}\right)$. However, this model is not practical. As *k* increases, (i) the number of observations decreases, (ii) the number of parameters increases, (iii) since most covariates take the same or similar values at t-1, t-2,…t-*k*, multicollinearity becomes a serious problem, and (iv) the portion of individuals stayed in the same company for long years increases; that may cause a sample selection bias.

Nawata (23, 57) evaluated the risk factors of HD and CBD; however, the histories of other diseases and interactions of medications were not considered. Since some individuals simultaneously take two or more different types of medications, interactions between medications are very important issues to consider. Their effects were also evaluated in these models. The following models (Models A, B and C) were considered in the analysis.

Basic model:

(2) $$\begin{array}{c} P[y_{t+1}=1|y_{t}=0]=\Lambda (\beta_{1}+\beta_{2}Age+\beta_{3}Female+\beta_{4}t1\;\\ +\;\beta_{5}BMI+\beta_{6}SBP+\beta_{7}DBP+\beta_{8}B\_Sugar+\beta_{9}HbA1c\;\\ +\;\beta_{10}HDL+\beta_{11}LDL+\beta_{12}Triglyceride+\beta_{13}ALT+\beta_{14}AST\;\\ +\;\beta_{15}GGP+\beta_{16}U\_Sugar+\beta_{17}U\_\mathrm{Pr}otein+\beta_{18}Smoke\;\\ +\;\beta_{19}Alcohol\_Freq+\beta_{20}Alcohol\_Amount+\beta_{21}Weight\_1\;\\ +\;\beta_{22}Weight\_20+\beta_{23}Eat\_Fast+\beta_{24}Late\_Supper\;\\ +\;\beta_{25}No\_Breakfast+\beta_{26}Exercise+\beta_{27}Activity+\beta_{28}Speed\;\\ +\;\beta_{29}Sleep+\beta_{30}M\_BP+\beta_{31}M\_Glu\cos e+\beta_{32}M\_Cholestrol\;\\ +\;\beta_{33}M\_BP\&GL+\beta_{34}M\_BP\&CH+\beta_{35}M\_GL\&CH\;\\ +\;\beta_{36}M\_BP\&GL\&CH+\beta_{37}z_{1t}+\beta_{38}z_{2t})\; \end{array}$$

Model A (HD model):

(3) $$y_{t+1}\;=\;HD_{t+1},y_{t}\;=\;HD_{t},z_{1t}\;=\;CBD_{t}\;\mathrm{and}\;z_{2t}\;=\;KD_{t}.$$

Model B (CBD model):

(4) $$y_{t+1}\;=\;CBD_{t+1},y_{t}=CBD_{t},z_{1t}=HD_{t}\;\mathrm{and}\;z_{2t}=KD_{t}.$$

Model C (KD model):

(5) $$y_{t+1}\;=\;KD_{t+1},y_{t}=KDD_{t},z_{1t}=HD_{t}\;\mathrm{and}\;z_{2t}=CBD_{t}$$

All covariates are values at t, and the subscript *t* is omitted in the covariates except *HD_t_, CBD_t_*, and *HD_t_*. The definitions of variables other than disease history are as follows:

*Age* (age of an individual),
*Female* (dummy variable) is 1 if female and 0 if male,
*Family* (dummy variable) is 1 if a family member and 0 otherwise,
*t1* (time trend) year – 2004,
*BMI* (body mass index) weight (kg)/[height (m)]^2^,
*SBP* (systolic blood pressure) mmHg,
*DBP* (diastolic blood pressure) mmHg,
*B_Sugar* (blood sugar) mg/dl,
*HbA1c* (hemoglobin A1c) %,
*HDL* (high-density lipoprotein cholesterol) mg/dl,
*LDL* (low-density lipoprotein cholesterol) mg/dl,
*Triglyceride* (serum triglyceride level) mg/dl,
*ALT* (alanine aminotransferase) units per liter (U/L),
*AST* (aspartate aminotransferase) U/L,
*GGP* (γ-glutamyl transferase) U/L,
*U_Sugar* (urine sugar; integers of 1–5) is 1 if undetected, 2 if around 50 mg/dl, 3 if around 100 mg/dl, 4 if around 250 mg/dl, and 5 if around 500 mg/dl or over,
*U_Protein* (urine protein; integers of 1–5) is 1 if undetected, 2 if around 15 mg/dl, 3 if around 30 mg/dl, 4 if around 100 mg/dl, and 5 if 250 mg/dl or over,
*Smoke* (dummy variable) is 1 if with smoking habit and 0 otherwise,
*Alcohol_Freq* (frequency of alcohol intake; integer 0–2) is 0 if never, 1 if sometimes, and 2 if every day,
*Alcohol_Amount* (amount of alcohol intake; integer 0–3) is 0 if none, 1 if drinking less than 180 ml of Japanese sake wine (with an alcohol percentage of about 15%) or equivalent alcohol per day when drinking, 2 if drinking 180–360 ml, 3 if drinking 360–540 ml, and 4 if drinking 540 ml or more,
*Weight_1* (dummy variable) is 1 if weight changed by 3 kg or more in a year and 0 otherwise
*Weight_20* (dummy variable) is 1 if weight increased by 10 kg or more from age 20 and 0 otherwise,
*Eat_Fast* (dummy variable) is 1 if eating faster than other people and 0 otherwise,
*Late_Supper* (dummy variable) is 1 if eating supper within two hours of bedtime three times or more in a week and 0 otherwise,
*No_Breakfast* (dummy variable) is 1 if not eating breakfast three times or more in a week and 0 otherwise,
*Exercise* (dummy variable) is 1 if exercising for 30 min or more twice or more in a week for more than a year and 0 otherwise,
*Activity* (dummy variable) is 1 if performing physical activities (walking or equivalent) for one hour or more daily and 0 otherwise,
*Speed* (dummy variable) is 1 if walking faster than other people of a similar age and the same gender and 0 otherwise, and
*Sleep* (dummy variable) is 1 if sleeping well and 0 otherwise.

In addition to these variables, the following dummy variables representing medication use were used to evaluate the side effects of medications.

*M_BP* (dummy variable) is 1 if taking antihypertensive medications to control BP only (not taking other types of medications) and 0 otherwise,
*M_Glucose* (dummy variable) is 1 if taking antihyperglycemic medications to control glucose (including insulin injections) only and 0 otherwise,
*M_Cholestrol* (dummy variable) is 1 if taking cholesterol medications to control cholesterol (including triglycerides) only and 0 otherwise,
*M_ BP & GL* (dummy variable) is 1 if taking two types of medications (antihypertensive and antihyperglycemic medications) and 0 otherwise,
*M_ BP & CH* (dummy variable) is 1 if taking two types of medications (antihypertensive and cholesterol medications) and 0 otherwise,
*M_ GL & CH* (dummy variable) is 1 if taking two types of medications (antihyperglycemic and medications) and 0 otherwise, and
*M_ BP & GL & CH* (dummy variable) is 1 if taking three types of medications (antihypertensive, antihyperglycemic, and cholesterol medications) and 0 otherwise.

**Figure 1:**
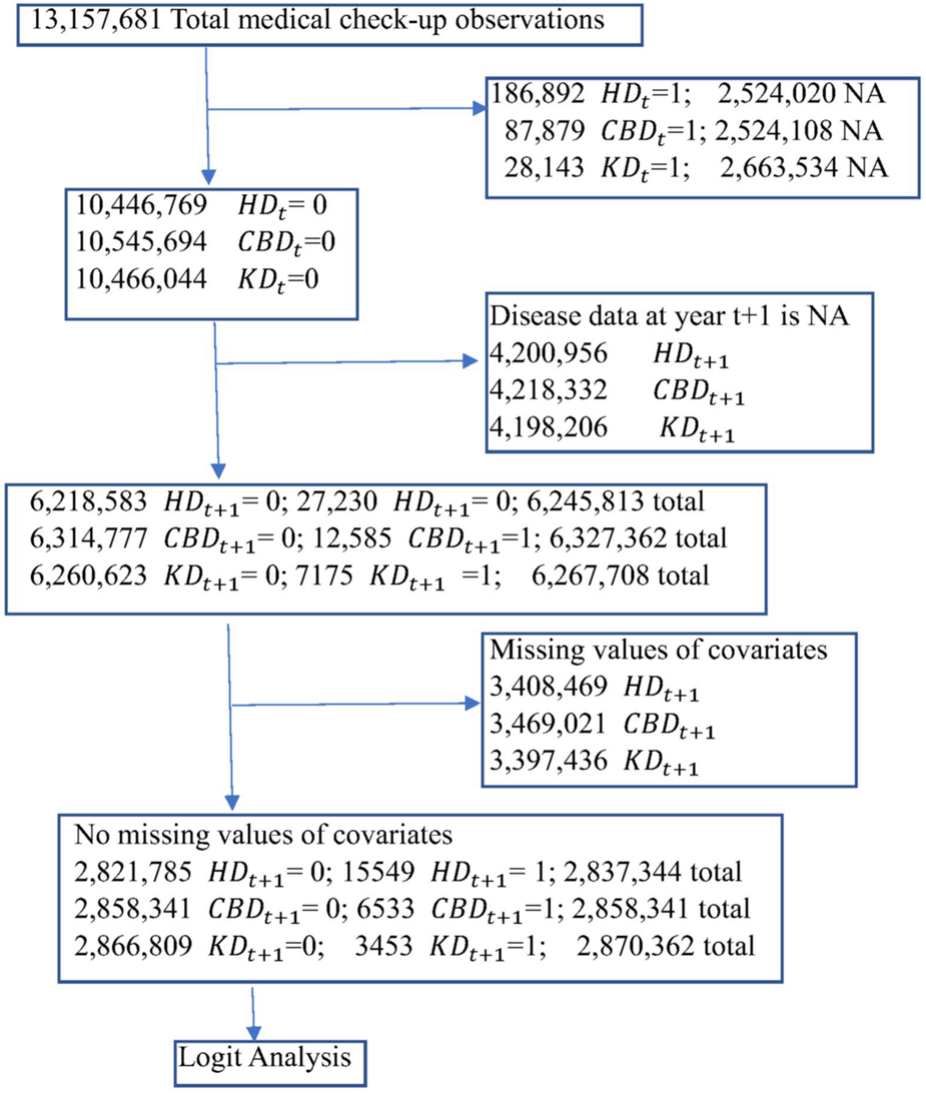
Flow chart of the study. NA, not available.

The estimation is done by the following steps. First, observations with $y_{t}\;=\;0$ and $y_{t+1}\;=\;0\;\mathrm{or}\;1\;$ are selected where $y_{t}$ is a variable representing a disease history. Then the observations with missing values of covariates are excluded. 2,837,334 (0:99.452%; 1:0.558%), 2,864,874 (0:99.771%; 1:0.299%) and 2,870,262 (0:99.876%; 1:0.120%) observations satisfy these criteria for Models A, B and C, respectively. The logit models are estimated using these observations. The design of the study is summarized in Figure 1. The summary of the covariates of 2,795,932 observations used in all models is given in Table 2. Table 7 in Appendix B is the list of abbreviations used in the study.

**Table 2:** Summary of covariates.

Variable	Average	SD	Variable	
*Age*	47.65	9.48	*Eeat_Fast*	1:32.5%; 0:67.5%
*Female*	0.38	0.04	*Late_Super*	1:32.3%; 0:67.7%
*Family*	0.22	0.05	*No_Breakfast*	1:17.9%; 0:82.1%
*t1*	11.01	2.05	*Exercise*	1:21.5%; 0:78.5%
*BMI*	22.95	3.64	*Activity*	1:34.9%; 0:65.1%
*SBP*	119.82	16.21	*Speed*	1:45.1%; 0:54.9%
*DBP*	74.40	11.81	*Weight_1*	1:26.0%; 0:74.0%
*B_Sugar*	95.42	18.13	*Weight_20*	1:35.1%; 0:64.9%
*HbA1C*	5.54	0.60	*Sleep*	1:59.0%; 0:41.0%
*HDL*	63.48	16.80	*M_BP*	1:6.94%; 0:93.06%
*LDL*	121.92	30.84	*M_Glucose*	1:1.12%; 0:98.88%
*Triglyceride*	108.14	85.86	*M_Cholestrol*	1:3.59%; 0:96.41
*ALT*	23.22	17.71	*M_BP&GL*	1:0.77%; 0:99.23%
*AST*	22.27	10.53	*M_BP&CH*	1:2.46%; 0:97.54%
*GGP*	38.08	45.04	*M_Gl&Ch*	0:0.55%; 99.45%
*U_Sugar*	1:97.88%; 2:0.45%; 3:0.52%; 4:0.38%; 5:0.76%		*M_BP&GL&CH*	0:0.70%; 1:99.30%
*U_Protein*	1:89.63%; 2:7.62%; 3:2.23%; 4:0.51%; 5:0.12%		*HD_t_*	1: 0.64%; 0:99.36%
*Smoke*	1:25.7%; 0:74.3%		*CBD_t_*	1:0.29%; 0:99.7%
*Drink_Freq*	1:40.69%; 1:34.06%; 2:25.25%		*KD_t_*	1:0.08%; 0:99.92%
*Drink_Amount*	0:40.69%; 1:22.14%; 2:22.78%; 3:10.62%; 4:3.77%		No. of observation	2,795,932

SD, standard deviation.

## 3. Results of estimation

The estimation results of Model A (HD model) are presented in Table 3. The gross percentage of HD patients in the following year was 0.55% among individuals without HD history. The estimate of *Age* was positive, and its *t*-value was quite large and significant at any reasonable significance level. The estimates for *Female, Family*, and *t1* were negative and significant at the 1% level. The estimates for *HbA1c, AST, U_Protein*, and *Weight_1* were positive and significant at the 1% level. The estimates of *HDL, LDL, ALT,* and *Sleep* were negative and significant at the 1% level. The estimates of *CBD_t_* and *KD_t_* were positive and significant at any reasonable level. The estimates of all dummy variables that represent medication use were positive, and their t-values were quite large and significant at any reasonable significance level. In particular, when antihypertensive medications were included, the estimated values were 0.657, 0.595, 0.793, and 0.784 for *M_BP*, *M_BP&GL, M_BP&CH*, and *M_BP&GL&CH*, respectively. These values were much larger than those obtained without antihypertensive medications.

**Table 3:** Results of estimation: model A (HD model).

Variable	Estimate	SE	Variable	Estimate	SE
*Constant*	−7.21115	0.13026	*Drink_Amount*	0.01947	0.01043
*Age*	0.03694	0.00106	*Eat_Fast*	0.01469	0.01760
*Female*	−0.27278	0.02796	*Late_Super*	0.03265	0.01835
*Family*	−0.09015	0.03156	*No_Breakfast*	0.02964	0.02285
*t1*	−0.00073	0.00414	*Exercise*	0.00139	0.02043
*BMI*	0.00394	0.00303	*Activity*	0.00866	0.01814
*SBP*	−0.00083	0.00082	*Speed*	−0.01543	0.01691
*DBP*	0.00007	0.00116	*Weight_1*	0.17872	0.01864
*B_Sugar*	0.00026	0.00060	*Weight_20*	0.03715	0.02014
*HbA1C*	0.07146	0.01901	*Sleep*	−0.12984	0.01670
*HDL*	−0.00360	0.00063	*M_BP*	0.65683	0.02505
*LDL*	−0.00125	0.00028	*M_Glucose*	0.23162	0.06673
*Triglyceride*	−0.00010	0.00010	*M_Cholesterol*	0.34615	0.03741
*ALT*	−0.00293	0.00078	*M_BP&GL*	0.59542	0.06273
*AST*	0.00306	0.00103	*M_BP&CH*	0.79275	0.03484
*GGP*	0.00023	0.00019	*M_Gl&CH*	0.25487	0.08759
*U_Sugar*	−0.02111	0.01747	*M_BP&GL&CH*	0.78428	0.06004
*U_Protein*	0.09283	0.01441	*CDB_t_*	0.54084	0.05167
*Smoke*	−0.02748	0.01956	*KD_t_*	0.53458	0.10516
*Drink_Freq*	−0.02622	0.015537			
Log likelihood	−96,464.1				
No. of observations, 0:28,21,785; 1:15,549; total:28,37,334					

SE, standard error.

Table 4 shows the results of the estimation of Model B (CBD model). The gross percentage of those with CBD in the following year was 0.23%. The estimate of *Age* was positive, and its *t*-value was quite large and significant at any reasonable significance level. The estimates for *DBP, GGP, U_Protein*, and *Weight_1* were positive and significant at the 1% level. In contrast, the estimates of *Speed* and *Sleep* were negative and significant at the 1% level and that of *LDL* was significant at the 5% level. The estimates of *HD_t_* and *KD_t_* were positive and significant at any reasonable level. The estimates of all dummy variables that represent medication use were positive, and their *t*-values were quite large and significant at any reasonable significance level. In particular, when antihypertensive medications were used, the estimated values were much higher than those without antihypertensive medications.

**Table 4:** Results of estimation: model B (CBD model).

Variable	Estimate	SE	Variable	Estimate	SE
Constant	−8.67888	0.20214	*Drink_Amount*	−0.01498	0.01655
*Age*	0.04091	0.00165	*Eat_Fast*	−0.01409	0.02728
*Female*	−0.06045	0.04223	*Late_Super*	0.05067	0.02847
*Family*	−0.07564	0.04677	*No_Breakfast*	0.06453	0.03529
*t1*	0.00191	0.00640	*Exercise*	0.00229	0.03153
*BMI*	−0.00850	0.00467	*Activity*	−0.00531	0.02803
*SBP*	0.00153	0.00123	*Speed*	−0.07967	0.02613
*DBP*	0.00754	0.00176	*Weight_1*	0.21776	0.02856
*B_Sugar*	−0.00009	0.00091	*Weight_20*	0.05532	0.03104
*HbA1C*	0.01128	0.02943	*Sleep*	−0.11694	0.02574
*HDL*	−0.00417	0.00097	*M_BP*	0.63846	0.03817
*LDL*	−0.00089	0.00043	*M_Glucose*	0.38296	0.10121
*Triglyceride*	−0.00003	0.00015	*M_Cholesterol*	0.50811	0.05367
*ALT*	−0.00243	0.00128	*M_BP&GL*	0.76571	0.09117
*AST*	0.00037	0.00188	*M_BP&CH*	0.79048	0.05148
*GGP*	0.00091	0.00027	*M_GL&CH*	0.48364	0.12565
*U_Sugar*	0.01009	0.02549	*M_BP&GL&CH*	0.77506	0.08914
*U_Protein*	0.12026	0.02103	*HD_t_*	0.76338	0.05129
*Smoke*	−0.01538	0.03050	*KD_t_*	0.77583	0.13258
*Drink_Freq*	−0.00789	0.024118			
Log likelihood	−44,642.38				
No. of observations, 0:28,58,341; 1:6,533; total:28,64,874					

SE, standard error.

The estimated results for Model C (KD model) are listed in Table 5. The percentage of patients with KD in the following year was 0.12%. Again, the estimate of *Age* was positive and its *t*-value was quite large and significant at any reasonable significance level. The estimate of *t1* was positive and significant at the 1% level, while that of *Female* was negative and significant at the 5% level. The estimates of *U_Protein* and *Weight_1* were positive and significant at the 1% level, and those of *U_Sugar* and *Late_Supper* were significant at the 5% level. The estimates of *BMI, HbA1c, ALT, Smoke, Drink_Freq*, and *Activity* were negative and significant at the 1% level. The estimates of *HD_t_* and *CBD_t_* were positive and significant at any reasonable significance level. As in the previous two models, the estimates of all dummy variables that represent medication use were positive and significant at any reasonable significance level. Especially, for the variables with antihypertensive medications, the estimates of *M_BP*, *M_BP&GL*, *M_BP&CH*, and *M_BP&GL&CH* were 1.01, 1.17, 1.30, and 1.27, respectively. These estimates were almost twice as large as those obtained without antihypertensive medications.

**Table 5:** Results of estimation: model C (KD model).

Variable	Estimate	SE	Variable	Estimate	SE
Constant	−7.09070	0.28123	*Drink_Amount*	−0.03316	0.02348
*Age*	0.01446	0.00221	*Eeat_Fast*	0.05439	0.03726
*Female*	−0.12230	0.05595	*Late_Super*	0.07855	0.03895
*Family*	−0.07391	0.06166	*No_Breakfast*	−0.02130	0.04847
*t1*	0.07559	0.00933	*Exercise*	0.04383	0.04408
*BMI*	−0.02806	0.00637	*Activity*	−0.12714	0.03911
*SBP*	−0.00323	0.00171	*Speed*	−0.06937	0.03615
*DBP*	0.00366	0.00241	*Weight_1*	0.18840	0.03912
*B_Sugar*	−0.00240	0.00128	*Weight_20*	−0.01114	0.04348
*HbA1C*	−0.20325	0.04227	*Sleep*	−0.04469	0.03538
*HDL*	−0.00223	0.00132	*M_BP*	1.01100	0.05304
*LDL*	−0.00054	0.00059	*M_Glucose*	0.56749	0.15760
*Triglyceride*	0.00097	0.00017	*M_Cholesterol*	0.47914	0.08520
*ALT*	−0.00769	0.00192	*M_BP&GL*	1.17028	0.12414
*AST*	0.00249	0.00271	*M_BP&CH*	1.30465	0.06793
*GGP*	−0.00111	0.00048	*M_Gl&CH*	0.65557	0.20017
*U_Sugar*	0.07924	0.03394	*M_BP&GL&CH*	1.27707	0.11667
*U_Protein*	0.80420	0.01704	*HD_t_*	0.45206	0.07604
*Smoke*	−0.15869	0.04313	*CBD_t_*	0.45799	0.10089
*Drink_Freq_F1*	−0.11585	0.034854			
Log likelihood	−25,006.4				
No. of observations, 0:28,66,809; 1:3,453; total:28,70,262					

SE, standard error.

## 4. Discussion

**Figure 2:**
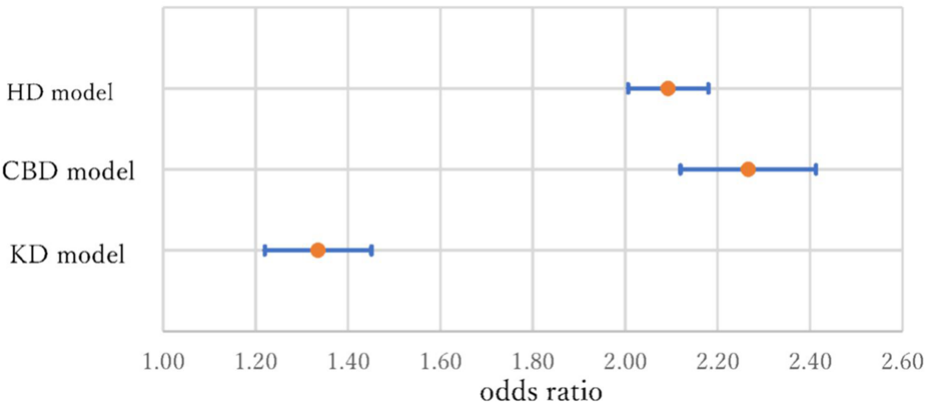
Odds ratios and 95% confidence intervals of *Age* (Age 50 vs. Age 70) in three models.

Table 6 summarizes the significant covariates other than disease histories and variables that represent taking medications in Models A (HD model), B (CBD model), and C (KD model). As expected, *Age* is a very important variable affecting these diseases. As shown in Figure 2, the odds ratios (ORs) comparing individuals aged 50–70 years were 2.09 with a 95% confidence interval (CI) of 2.01–2.18 for HD, 2.27 with a 95% CI of 2.12–2.41 for CBD, and 1.34 with a 95% CI of 1.22–1.45 for KD. Although Nawata (23) evaluated the risk factors for HD, the ORs and CIs were not evaluated. Since the probabilities of having these diseases in the next year are small, OR and CI are approximately equal to the probability ratio (PR) and its CI (57). The risk of HD and CBD in individuals aged 70 years is more than twice as large as those of individuals aged 50 years. The increase in risk was relatively small for KD. Female is significant at the 1% level in Model A but not significant at the 5% level in other models.

**Table 6:** Significant variables and the signs of estimates in three models.

	Model A (HD model)		Model B (CBD model)		Model C (KD model)	
	Positive	Negative	Positive	Negative	Positive	Negative
*Age*	**		**		**	
*Female*		**				
*Family*		**				**
*t1*			**		**	
*BMI*					**	
*HbA1c*	**					**
*HDL*		**		**		
*LDL*		**		**		
*ALT*		**				**
*AST*	**					
*GGP*			**			**
*U_Protein*	**		**		**	
*U_Sugar*					**	
*Late_Supper*						
*Activity*						**
*Weight_1*	**		**		**	
*Sleep*		**		**		

* Significant at the 5% level.

** Significant at the 1% level.

**Figure 3:**
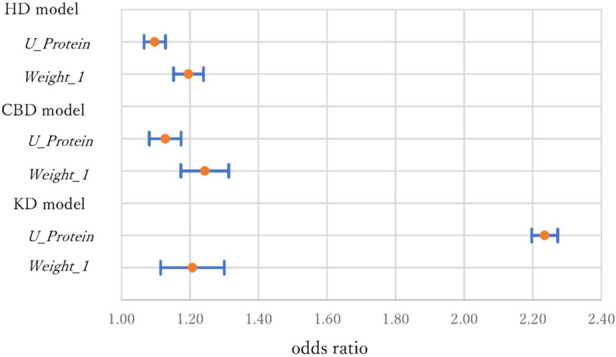
Odds ratios and 95% confidence intervals of *U_Protein* and *Weight_1* in three models.

*Weight_1* (recent weight change) and *U_Protein* (urine protein level) were significant at the 1% level in all models and were considered important variables. Figure 3 shows the ORs and 95% CIs of these variables for the models. For the dummy variable, the OR was calculated by comparing 0 and 1. For *U_Protein*, the majority of the values were 1 or 2, so the OR was calculated by comparing *U_Protein* = 1 and 2. For HD, the ORs of *U_Protein* and *Weight_1* (95% CI) were 1.10 (1.0–1.13) and 1.20 (1.15–1.24.) For CBD, the ORs were1.13 (1.08–1.17) and 1.24 (1.17–1.31). For KD, the ORs were 2.23 (2.20–2.31) and 1.21 (1.11–1.30). *Weight_1* increased the risk by approximately 20% for all diseases. *U_Protein* increased the risk by about 10% for HD and CD; however, it doubled the risk for KD. *U_Protein* is a risk factor with obvious mechanisms assumed (58) and people with high urine protein levels should recognize this fact, and special attention is necessary.

Another interesting finding is that among BP levels (*SBP* and *DBP*), only *DBP* in the CBD model was significant, and the estimates were not significant in the other models. This may raise questions regarding the 2017ACC/AHA guideline, which lowered the hypertensive criterion.

**Figure 4:**
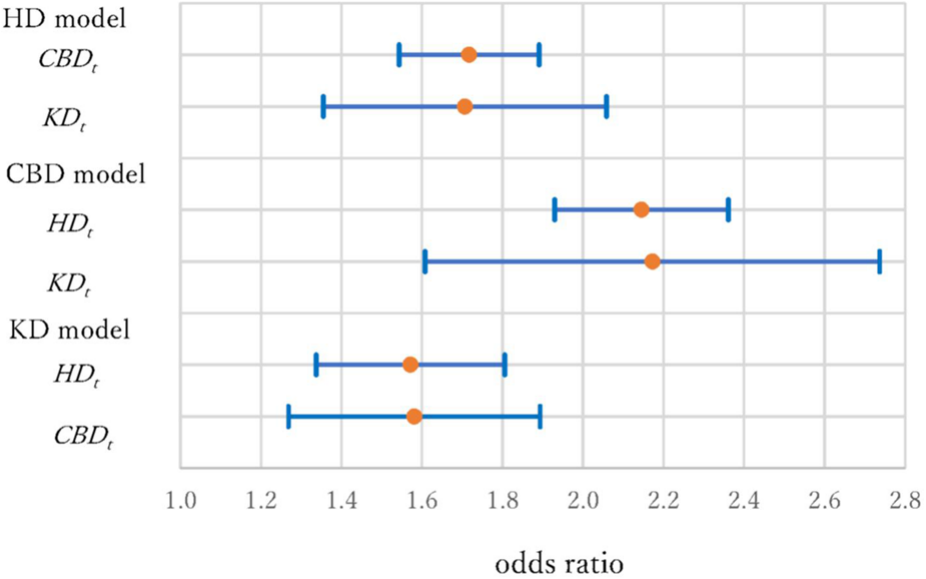
Odds ratios and 95% confidence intervals of disease histories (*HD_t_, CBD_t_* and *KD_t_*) in three models.

Figure 4 shows the ORs and 95% CIs for the variables of a history of other diseases. In the HD model, the ORs were 1.72 (1.54–1.89) and 1.71 (1.35–2.06) for *CBD_t_* and *KD_t_*. In the CBD model, the ORs were 2.15 (1.93–2.36) and 2.17 (1.61–2.74) for *HD_t_* and *KD_t_*. In the KD model, the ORs were 1.57 (1.34–1.81) and 1.58 (1.27–1.89) for *HD_t_* and *CBD_t_*. The risk of having CBD for individuals with HD and KD histories was especially high, more than double of those without disease histories, and special care is necessary for them (57).

**Figure 5:**
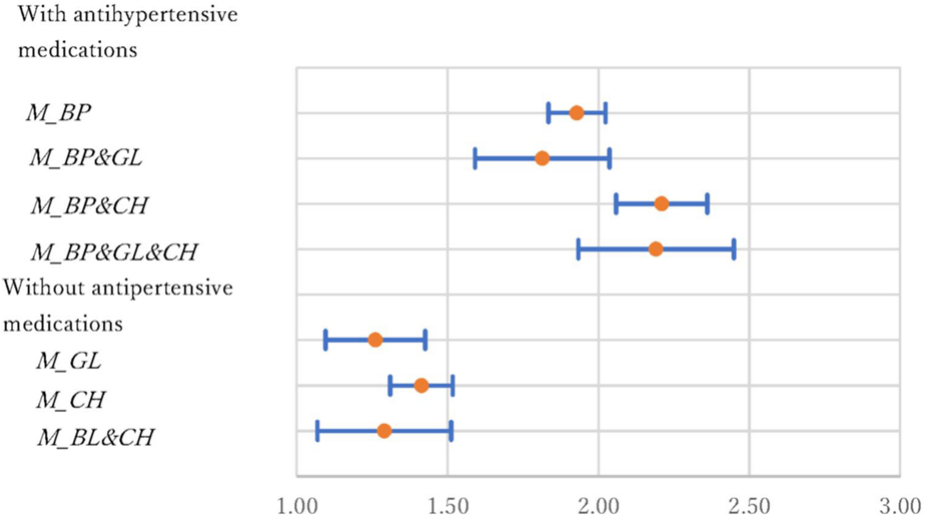
Odds ratios and 95% confidence intervals of medications in the HD model.

**Figure 6:**
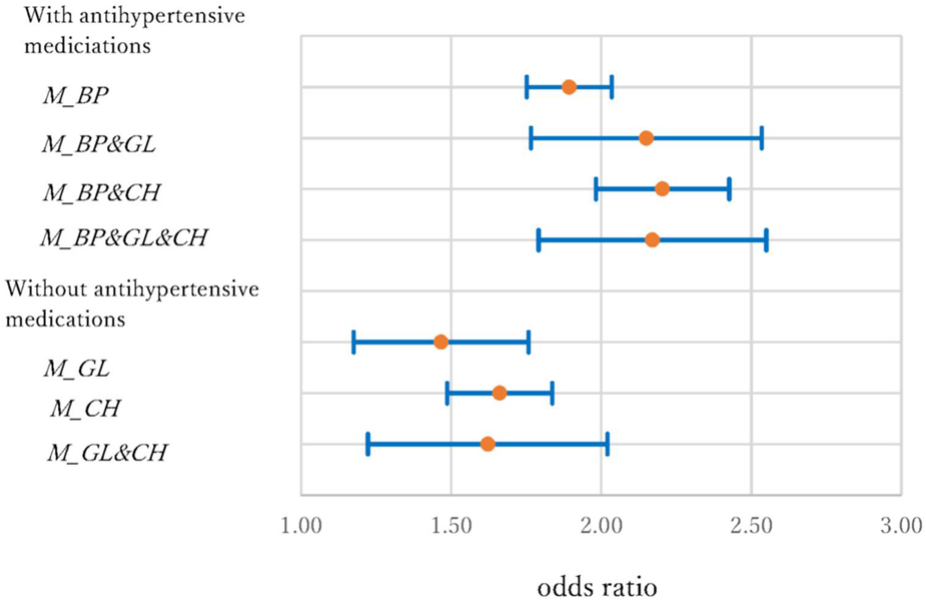
Odds ration and 95% confidence interval of medications in the CBD model.

**Figure 7:**
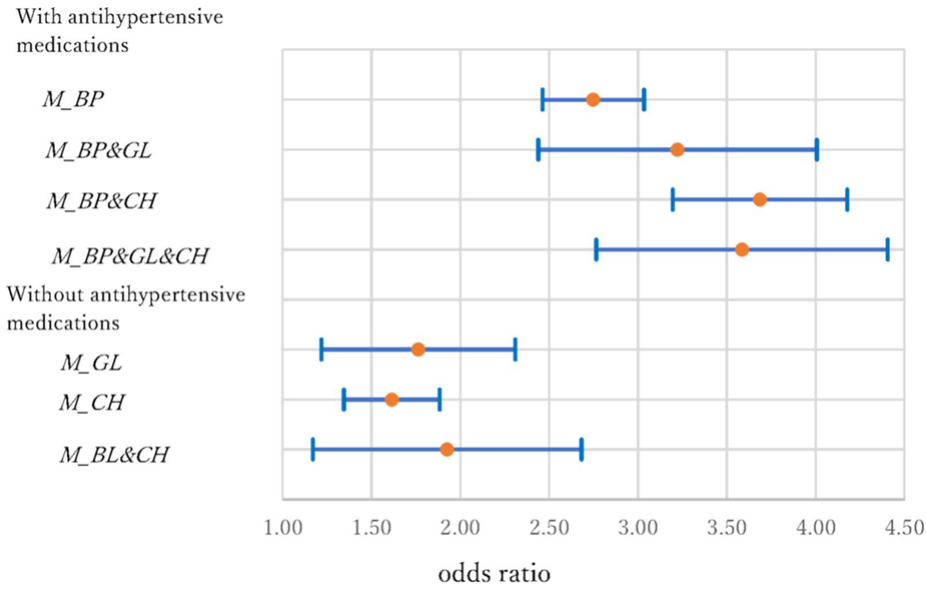
Odds ratios and 95% confidence intervals of medications in the KD model.

All medications (antihypertensive, antihyperglycemic, and cholesterol-lowering) increased the risk of all three diseases. Figures 5–7 show ORs and 95% CIs, respectively. People often take two or three types of medications, and seven different variables are separately shown in the figures. These variables were divided into two groups. One group was treated with antihypertensive medications (i.e., M_BP, *M_BP&GL, M_BP&CH,* and *M_BP&GL&CH*), and the other was without antihypertensive medications (i.e., *M_GL, M_CH*, and *M_GL&CH*). In the HD model, the ORs (95% CI) were 1.93 (1.83–2.02), 1.81 (1.59–2.04), 2.21 (2.06–2.36), and 2.19 (1.93–2.45) for *M_BP, M_BP&GL, M_BP&CH*, and *M_BP&GL&CH*, respectively. In contrast, the ORs were 1.26 (1.10–1.43), 1.41 (1.31–1.52), and 1.29 (1.07–1.51) for *M_GL, M_CH,* and *M_GL&CH*. In the former group, the risks associated with antihypertensive medications were almost double compared with those without medications. In the latter group, the increments were much smaller, approximately 25%–40%.

In the CBD model, the ORs (95% CI) were 1.89 (1.75–2.04), 2.15 (1.77–2.53), 2.20 (1.98–2.43), and 2.17 (1.79–2.55) for *M_BP, M_BP&GL, M_BP&CH,* and *M_BP&GL&CH*, respectively. In contrast, the ORs were 1.47 (1.18–1.86), 1.66 (1.49–1.84), and 1.62 (1.22–2.02) for *M_GL, M_CH,* and *M_GL&CH*. In the former group, the risks associated with medications were almost double compared to those without medication, as in the case of heart disease. In the latter group, the increments were about 50%–65%. The results of this study suggest that antihypertensive medications increase the risks of HD and CBD, and the statement of Muntner et al. (11) is not supported.

Concerning the KD model, the ORs (95% CI) were 2.75 (2.46–3.93), 3.22 (2.44–4.01), 3.69 (3.20–4.18), and 3.59 (2.77–4.41) for *M_BP, M_BP&GL, M_BP&CH,* and *M_BP&GL&CH,* respectively. In contrast, the ORs were 1.76 (1.22–2.31), 1.61 (1.35–1.88), and 1.93 (1.17–2.68) for *M_GL, M_CH,* and *M_GL&CH*. In the KD mode1, although the CIs were larger, the increments in the risks seem much higher than those of the previous two diseases. The risks triple (or increase more) in the first group. The increments were about 60%–70% in the second group. Especially in the first group, even the lower bounds of the 95% CIs, the risks become 2.4–3.2 times larger than those without medications. This may imply that the negative effects of antihypertensive medications are much more serious in patients with KD.

The interactions between medications are important issues when individuals use two or more different types of medications. In the models without antihypertensive medications, the 95% CIs of *M_GL* and *M_CH* overlapped with the CI of *M_GL&CH*, and no significant interaction effect was observed. However, when antihypertensive and cholesterol medications were used simultaneously (*M_ BP & CH*), the risks were significantly higher (i.e., the 95% CIs did not overlap) than those using only antihypertensive medications (*M_BP*) in the HD and KD models.

Not only are the values of estimates of antihypertensive medications much higher than those of the other medications but the use of antihypertensive medications is also higher than that of other medications (antihypertensive medications: 11.18%, antihyperglycemic medications: 3.21%, and cholesterol medications: 7.46%). Here, we mainly discuss antihypertensive medications. For possible interpretations of the positive estimates, see Nawata (23).

Although there are some alternative therapies such as renal denervation (59), prescribing antihypertensive medications is a major therapy for hypertension. Over 200 drugs are available globally (60). Jackson and Bellamy (61) classified these medications into two group. One group is being those which directly or indirectly block the renin–angiotensin system (RAS) and the other group works through non-RAS pathways. The first group includes angiotensin-converting enzyme inhibitors (ACEIs), angiotensin-converting enzyme inhibitors, angiotensin receptor antagonists and direct renin inhibitors. The second group consists of adrenoceptor antagonists (β-blockers, α-blockers), calcium channel blockers, diuretics (thiazides, loop, potassium sparing/aldosterone antagonist), vasodilators, centrally acting agents, and ganglion block.

Laurent (62) also classified the medications into β-blockers, diuretics, ACEIs, angiotensin II receptor blockers (ARBs), calcium-channel blockers, and other classes. Although the information of individual prescription records is not available, ACIs and ARAs are most widely used antihypertensive medications in Japan (63). RAS is a regulator of blood volume, electrolyte balance, and systemic vascular resistance. For summaries of RAS in the management of hypertension, see Fountain et al. (64) and Lange-Jacobs et al. (65). RAS is an extreme complex system in playing hypertension, and the understanding of RAS has expanded tremendously over the last few decades (65). Crifciler and Haznedarouglu (66) outlined intersections of circulating and local angiotensin systems in the vascular pathobiological microenvironment of central nervous system.

Since antihypertensive medications are widely used, their mechanisms of action, effectiveness, proper usage methods, and side effects have been widely studied (67–76). Lithell (67) reported that “both β-blockers and thiazide diuretics worsened insulin resistance and deteriorated lipoprotein metabolism. …These data may explain the unexpectedly high incidence of diabetes among hypertensive patients and the poor effect on risk for coronary heart disease in intervention trials.” Santos et al. (68) did systematic reviews to identify the possible pharmacogenetic implications for RAS-blocker medications in the hypertension-CKD scenario. They described that studies focusing on CKD were scarce. The CDC (77) and International Diabetes Foundation (78) described KD as a serious diabetes complication.

Marcum and Fried (79) mentioned that antihypertensive medications are frequently used in older adults with CKD, and the most common adverse drug events (ADEs) with antihypertensive use include acute kidney injury and antihypertensives, which may lead to medication errors and ADEs. Even in the Systolic Blood Pressure Intervention Trial (SPRINT) (16), which was heavily weighted in the 2017ACC/AHA guideline, it was admitted that “Rates of serious adverse events of hypotension, syncope, electrolyte abnormalities, and acute kidney injury or failure, …, were higher in the intensive-treatment group than in the standard-treatment group.” They also reported the percentages of the serious adverse events in both groups. These findings are consistent with the results of the present study. Although Ptinopoulou et al. (80) wrote that “it is now common knowledge that adequate blood pressure control is the most important factor for the preservation of renal function, so every drug that effectively lowers hypertension is believed to be renoprotective,” the findings of the paper do not support this statement. The risks of hypertensive medications were three times or more higher than those without medications for KD. Any medication may have negative side effects. In particular, the risk of KD is much higher for individuals taking antihypertensive medications. These medications must be prescribed with caution to minimize any negative side effect, and further studies are needed on the risks of antihypertensive medications.

We obtained slightly different results from those of previous studies. One possible reason might be a sample selection bias. In this study, we analyzed the general population. In other studies, special individuals with a high risk of diseases were analyzed. The rate of having heart diseases in the next year is about 0.5%. If the sample size is 10 thousand, the expected number of new patients is just 50 per year and it is sometimes difficult to get statistically significant results. In the SPRINT (16), they selected 9,361patients (4,678: intensive treatment; 4,683: standard treatment) at high risk for cardiovascular events. They admitted that “The lack of generalizability to populations … is a limitation.” Over 2.8 million observations were used in the study and the generalizability problem might be improved. For the theoretical details, see Nawata and Kimura (19).

## 5. Conclusion

In this study, the risk factors for heart, cerebrovascular, and kidney diseases (HD, CBD, and KD) were analyzed using data from 2,837,334, 2,864,874, and 2,870,262 medical checkups obtained from the JMDC claims database. The data of individuals who had no history of each disease at year t and had information in the following year were analyzed using the logit models. Among the health-related information, age was a very important risk factor. The histories of other types of diseases were also very important factors, and the risks of HD, CBD, and KD would increase when individuals had histories of other diseases.

Among other factors, urine protein levels and recent large weight changes were very important for all three diseases. For KD, the risk would be more than double for individuals with high urine protein levels, and these individuals should be aware of this fact to prevent KD.

The side effects of the medications, including interactions, were also evaluated. Taking antihypertensive medications increased the risk of disease. The negative side effects were especially severe when individuals were taking antihypertensive drugs. Antihypertensive medications are widely used. Although the 2017ACC/AHA guideline lowered the hypertensive criterion, special care and additional studies are necessary to prescribe these medications, particularly antihypertensive medications so as to minimize the negative side effects of medications.

As the dataset comprises the health checkups of workers in Japan, it does not include individuals aged 76 and above. Age is a very important factor in these diseases, and the risk of these diseases increases with age. Although no precise data are not available, it is indicated that adequate care and consideration should be given to antihypertensive medications for the elderly. It is also necessary to collect data on older adults. The dataset only contained information obtained in Japan. The Japanese are ethnically homogeneous, and potential ethnic effects on the diseases are not evaluated. This might be an advantage to examine factors such as effects of medications because underlying genetic influences might be small. However, we may get different results in other countries, and that is a limitation of this study. Further studies are needed for generalization of this study. These will be the subjects of future studies.

## Data Availability

Publicly available datasets were analyzed in this study. This data can be found here: JMDC Claims Database https://www.jmdc.co.jp/en/jmdc-claims-database/.

## APPENDIX

Appendix A. Confidence intervals

The confidence interval of the ratio of the two estimators is calculated using the following formula:

(A.1) $$\frac{\hat{a}}{\hat{b}}\;=\;\frac{\hat{a}}{\hat{b}}-\cdot -\frac{\hat{a}}{b}+\frac{\hat{a}}{b}-\frac{a}{b}\;=\;\hat{a}\left(\frac{1}{\hat{b}}-\frac{1}{b}\right)\;+\;\frac{1}{b}(\hat{a}-a)$$

(A.2) $$\hat{a}\left(\frac{1}{\hat{b}}-\frac{1}{b}\right)\;=\;\hat{a}\frac{b-\hat{b}}{b\hat{b}}\;=\;\frac{a}{b^{2}}(b-\hat{b})\;+\;o_{p}\left(n^{-\frac{1}{2}}\right).$$

From (A.1) and (A.2), we get

(A.3) $$\frac{\hat{a}}{\hat{b}}\;-\;\frac{a}{b}\;=\;\frac{1}{b}(\hat{a}-a)\;+\;\frac{a}{b^{2}}(b-\hat{b})\;+\;o_{p}\left(n^{-\frac{1}{2}}\right)\begin{array}{c} A\\ ∼ \end{array}N(0,\;\omega )$$

$$\omega \;=\;(\frac{1}{b}{)}^{2}V(\hat{a})\;+\;(\frac{a}{b^{2}}{)}^{2}V(\hat{b}).$$

Appendix B. Abbreviations

The following table is the list of abbreviations used in this paper.

**Table 7:** List of abbreviations (symbols and their meaning).

Symbol	Meaning	Symbol	Meaning
HD	heart disease	*AST*	aspartate aminotransferase
CBD	cerebrovascular disease	*GGP*	γ-glutamyl transferase
KD	kidney disease	*U_Sugar*	urine sugar
CVD	cardiovascular disease	*U_Protein*	urine protein
IHD	ischemic HD	*Alcohol_Freq*	frequency of alcohol intake
CKD	chronic KD	*Alcohol_Amount*	amount of alcohol intake
*HD_t_*	having a HD history at t	*Weight_1*	weight change in a year
*CBD_t_*	having a CVD history at t	*Weight_20*	weight change from age 20
*KD_t_*	having a KD history at *t*	*Eat_Fast*	eating faster
*Age*	age of a person	*Late_Supper*	late supper
*Female*	gender	*No_Breakfast*	not eating breakfast
*family*	family member	*Exercise*	exercising
*t1*	time trend	*Activity*	doing physical activities
*BMI*	body mass index	*Speed*	walking faster
*SBP*	systolic blood pressure	*Sleep*	sleeping well
*DBP*	diastolic blood pressure	*M_BP*	taking antihypertensive medications
*B_Sugar*	blood sugar	*M_Glucose*	taking antihyperglycemic medications
*HbA1c*	hemoglobin A1c	*M_Cholestrol*	taking cholesterol medications
*HDL*	high-density lipoprotein cholesterol	*M_ BP & GL*	taking antihypertensive and antihyperglycemic medications
*LDL*	low-density lipoprotein cholesterol	*M_ BP & CH*	taking antihypertensive and cholesterol medications
*Triglyceride*	serum triglyceride level	*M_ GL & CH*	taking antihyperglycemic and medications
*ALT*	alanine aminotransferase	*M_ BP & GL & CH*	taking antihypertensive, antihyperglycemic, and cholesterol medications

## References

[1] World Heath Organization (WHO). “The top 10 causes of death.” (2020). Available at: https://www.who.int/news-room/fact-sheets/detail/the-top-10-causes-of-death

[2] National Center for Health Statistics (NCHS, 2021). “Mortality in the United States, 2020,” *NCHS Data Brief*, Number 427. Available at: https://www.cdc.gov/nchs/data/databriefs/db427-tables.pdf#4

[3] Ministry of Health, Labour and Welfare. “Reiwa 2 nen jinnkou doutai toukei no gaikyou (Summary of demographic statistics in 2020)” in Japanese (2022).

[4] Ministry of Health, Labour and Welfare. “Reiwa ichi nendo kokumin iryouhi no gaikyou (Estimates of national medical care expenditure, FY 2019)” in Japanese (2021).

[5] American Heart Association (AHA). “Understand your risks to prevent a heart attack.” (2016). Available at: https://www.heart.org/en/health-topics/heart-attack/understand-your-risks-to-prevent-a-heart-attack

[6] Centers for Disease Control and Prevention (CDC, 2022). “Heart disease and stroke.” Available at: https://www.cdc.gov/chronicdisease/resources/publications/factsheets/heart-disease-stroke.htm#:∼:text=Leading%20risk%20factors%20for%20heart,unhealthy%20diet%2C%20and%20physical%20inactivity

[7] Fuchs FD Whelton PK. High blood pressure and cardiovascular disease. Hypertension. (2020) 75:285–92. 10.1161/HYPERTENSIONAHA.119.14240 31865786 PMC10243231

[8] Whelton PK Carey RM Arrow WS Casey DE Collins KJ Himmelfarb CD 2017 ACC/AHA/AAPA/ABC/ACPM/AGS/APhA/ASH/ASPC/NMA/PCNA guideline for the prevention, detection, evaluation, and management of high blood pressure in adults: a report of the American college of cardiology/American heart association task force on clinical practice guidelines. Hypertension. (2018) 71:e13–e115. 10.1161/HYP.0000000000000065 29133356

[9] Chobanian AV Bakris GL Black HR Cushman WC Green LA Izzo JL Seventh report of the joint national committee on prevention, detection, evaluation, and treatment of high blood pressure. Hypertension. (2003) 42:1206–52. 10.1161/01.HYP.0000107251.49515.c2 14656957

[10] Nawata K. An analysis of blood pressure situations in Japan using the large-scale medical checkup dataset. Health. (2021) 13:736–56. 10.4236/health.2021.137057

[11] Muntner P Carey RM Gidding S Jones DW Taler SJ Wright JT Potential U.S. population impact of the 2017 ACC/AHA high blood pressure guideline. J Am Coll Cardiol. (2018) 71:109–18. 10.1016/j.jacc.2017.10.073 29146532 PMC5873591

[12] Lewington S Clarke R Qizilbash N Peto R Collins R, Prospective studies collaboration. age-specific relevance of usual blood pressure to vascular mortality: a meta-analysis of individual data for one million adults in 61 prospective studies. Lancet. (2002) 360:1903–13. 10.1016/S0140-6736(02)11911-8 12493255

[13] Cushman WC Evans GW Byington RP Goff DC Grimm RH Cutler JA Effect of intensive blood-pressure control in type 2 diabetes mellitus. N Engl J Med. (2010) 362:1575–85. 10.1056/NEJMoa1001286 20228401 PMC4123215

[14] Asayama K Satoh M Murakami Y Ohkubo T Nagasawa S Tsuji I Cardiovascular risk with and without antihypertensive drug treatment in the Japanese general population participant-level meta-analysis. Hypertension. (2014) 63:1189–97. 10.1161/HYPERTENSIONAHA.113.03206 24637661

[15] Rapsomaniki E Timmis A George J Pujades-Rodriguez M Shah AD Denaxas S Blood pressure and incidence of twelve cardiovascular diseases: lifetime risks, healthy life-years lost, and age-specific associations in 1.25 million people. Lancet. (2014) 383:1899–911. 10.1016/S0140-6736(14)60685-1 24881994 PMC4042017

[16] Wright JT Williamson JD Whelton PK Snyder JK Sink KM Rocco MV A randomized trial of intensive versus standard blood-pressure control. N Engl J Med. (2015) 373:2103–16. 10.1056/NEJMoa1511939 26551272 PMC4689591

[17] Ettehad D Emdin CA Kiran A Anderson SG Callender T Emberson J Blood pressure lowering for prevention of cardiovascular disease and death: a systematic review and meta-analysis. Lancet. (2016) 387:957–67. 10.1016/S0140-6736(15)01225-8 26724178

[18] Nawata K Kimura M. Does high systolic blood pressure truly increase medical expenditure? An empirical analysis of the new 2017 ACC/AHA hypertension guideline. Health. (2018) 10:1044–65. 10.4236/health.2018.108079

[19] Nawata K Kimura M. Empirical studies of effects of high blood pressure on medical costs and heart disease: is the 2017 ACC/AHA guideline supported by enough evidence? Health. (2018) 10:1498–519. 10.4236/health.2018.1011115

[20] Nawata K Sugano H Kimura M. An analysis of the effects of blood pressure and antihypertensive drugs on heart disease. Health. (2019) 11:792–816. 10.4236/health.2019.116064

[21] National Institute of Health. “The framingham heart study: laying the foundation for preventive health care.” (2021). Available at: https://www.nih.gov/about-nih/what-we-do/impact-nih-research/our-stories

[22] Saiz LC Gorricho J Garjón J Celaya MC Erviti J Leache L Blood pressure targets for the treatment of people with hypertension and cardiovascular disease,”. Cochrane Database Syst Rev. (2020) 9:CD010315. 10.1002/14651858.CD010315.pub4 32905623 PMC8094921

[23] Nawata K. Heart diseases, hypertension and effects of antihypertensive medications: is hypertension a true risk factor of heart diseases? Front Public Health. (2022) 10:929840. 10.3389/fpubh.2022.929840 36388284 PMC9659607

[24] American Stroke Association (ASA, 2021). “Stroke risk factors.” Available at: https://www.stroke.org/en/about-stroke/stroke-risk-factors

[25] Winstein CJ Stein J Arena R Bates B Cherney LR Cramer SC Guidelines for adult stroke rehabilitation and recovery, a guideline for healthcare professionals from the American heart association/American stroke association. Stroke. (2016) 47:e98–e169. 10.1161/STR.0000000000000098 27145936

[26] Gittler M Davis AM. Guidelines for adult stroke rehabilitation and recovery. JAMA. (2018) 319:820–1. 10.1001/jama.2017.22036 29486016

[27] Powers WJ Rabinstein AA Ackerson T Adeoye OM Bambakidis NC Becker K “2018 guidelines for the early management of patients with acute ischemic stroke: a guideline for healthcare professionals from the American heart association/American stroke association,”. Stroke. (2018) 49:e46–e110. 10.1161/STR.0000000000000158 29367334

[28] Powers WJ Rabinstein AA Ackerson T Adeoye OM Bambakidis NC Becker K Guidelines for the early management of patients with acute ischemic stroke: 2019 up-date to the 2018 guidelines for the early management of acute ischemic stroke: a guideline for healthcare professionals from the American heart association/American stroke association. Stroke. (2019) 50:e344–e418. 10.1161/STROKEAHA.118.022606 31662037

[29] George MG. Risk factors for ischemic stroke in younger adults, a focused update. Stroke. (2020) 51:729–35. 10.1161/STROKEAHA.119.024156 32078487 PMC7112557

[30] Stein J Katz DI Schaffer MB Cramer SC Deutsch AF Harvey RL Clinical performance measures for stroke rehabilitation performance measures from the American heart association/American stroke association. Stroke. (2021) 52:e675–e700. 10.1161/STR.0000000000000388 34348470

[31] Frerich S Malik R Georgakis MK Sinner MF Kittner SJ Mitchell BD Cardiac risk factors for stroke: a comprehensive Mendelian randomization study. Stroke. (2022) 53:e130–5. 10.1161/STROKEAHA.121.036306 34911345 PMC10510836

[32] CDC. “CKD risk factors and prevention.” (2022). Available at: https://www.cdc.gov/kidneydisease/publications-resources/annual-report/ckd-risk-prevention.html

[33] American Kidney Fund. “Risk factors.” (2022). Available at: https://www.kidneyfund.org/all-about-kidneys/risk-factors

[34] Kidney Foundation of Canada. “Risk factors.” (2022). Available at: https://kidney.ca/Kidney-Health/Newly-Diagnosed/Risk-Factors

[35] GBD Chronic Kidney Disease Collaboration. Global, regional and national burden of chronic kidney disease, 1990–2017: a systematic analysis for the global burden of disease study 2017. Lancet. (2020) 395:709–33. 10.1016/S0140-6736(20)30045-3 32061315 PMC7049905

[36] Sarnak MJ Amann K Bangalore S Cavalcante JL Charytan DM Craig JC Chronic kidney disease and coronary artery disease: JACC state-of-the-art review. J Am Coll Cardiol. (2019) 74:1823–38. 10.1016/j.jacc.2019.08.1017 31582143

[37] Hisatome I Li P Miake J Mahati E Maharani N Utami SB Uric acid as a risk factor for chronic kidney disease and cardiovascular disease – Japanese management of asymptotic hyperuricemia-. Circ J. (2021) 85:130–8. 10.1253/circj.CJ-20-0406 33342914

[38] Hashimoto Y Hamaguchi M Okamura T Nakanishi N Obora A Kojima T Metabolic associated fatty liver disease is a risk factor for chronic kidney disease. J Diabetes Investig. (2022) 13:308–16. 10.1111/jdi.13678 34561962 PMC8847128

[39] Kovesdy CP. Epidemiology of chronic kidney disease: an update 2022. Kidney Int Suppl. (2022) 12:7–11. 10.1016/j.kisu.2021.11.003 PMC9073222 35529086

[40] Wyld MLR De La Mata NL Viecelli A Swaminathan R O’Sullivan KM O'Lone E Sex-based differences in risk factors and complications of chronic kidney disease. Semin Nephrol. (2022) 42:153–69. 10.1016/j.semnephrol.2022.04.006 35718363

[41] Turner JM Bauer C Abramowitz MK Melamed ML Hostetter TH. Treatment of chronic kidney disease. Kidney Int. (2012) 81:351–62. 10.1038/ki.2011.380 22166846

[42] Liyanage T Ninomiya T Jha V Neal B Patrice HM Okpechi I Worldwide access to treatment for end-stage kidney disease: a systematic review. Lancet. (2015) 385:1975–82. 10.1016/S0140-6736(14)61601-9 25777665

[43] Heerspink HJL Greene T Tighiouarte H Gansevoort RT Coresh J Simon AL Change in albuminuria as a surrogate endpoint for progression of kidney disease: a meta-analysis of treatment effects in randomised clinical trials. Lancet Diabetes Endocrinol. (2019) 7:128–39. 10.1016/S2213-8587(18)30314-0 30635226

[44] Tinti F Lai S Noce A. Chronic kidney disease as a systemic inflammator syndrome: update on mechanisms involved and potential treatment. Life. (2021) 11:419. 10.3390/life11050419 34063052 PMC8147921

[45] Yamazaki T Mimura I Tanaka T Nangaku M. Treatment of diabetic kidney disease: current and future. Diabetes Metab J. (2021) 45:11–26. 10.4093/dmj.2020.0217 33508907 PMC7850867

[46] Abdelazeem B Shehata J Abbas KS El-Shahat NA Malik B Savarapu P The efficacy and safety of roxadustat for the treatment of anemia in non-dialysis dependent chronic kidney disease patients: an updated systematic review and meta-analysis of randomized clinical trials. PLoS One. (2022) 17:e0266243. 10.1371/journal.pone.0266243 35363823 PMC8974992

[47] YaghoubiFard S Goudarzi R Etminan A Baneshi M Barouni M Sirizi JM. Cost-effectiveness analysis of dialysis and kidney transplant in patients with renal impairment using disability adjusted life years in Iran. Med J Islam Repub Iran. (2016) 30:390. 27493934 PMC4972066

[48] Jarl J Desatnik P Hansson UP Prütz KG Gerdtham U. Do kidney transplantations save money? A study using a before–after design and multiple register-based data from Sweden. Clin Kidney J. (2018) 11:283–8. 10.1093/ckj/sfx088 29644072 PMC5888588

[49] Helanterä I Isola T Lehtonen TK Åberg F Lempinen M Isoniemi H. Association of clinical factors with the costs of kidney transplantation in the current era. Ann Transplant. (2019) 24:393–400. 10.12659/AOT.915352 31263093 PMC6625575

[50] Fu R Sekercioglu N Berta W Coyte PC. Cost-effectiveness of deceased-donor renal transplant versus dialysis to treat end-stage renal disease: a systematic review. Transplant Direct. (2020) 6:e522. 10.1097/TXD.0000000000000974 32095508 PMC7004633

[51] Kiberd BA Tennankore KK Vinson AJ. Comparing the net benefits of adult deceased donor kidney transplantation for a patient on the preemptive waiting list vs a patient receiving dialysis. JAMA Network Open. (2022) 5:e2223325. 10.1001/jamanetworkopen.2022.23325 35867058 PMC9308061

[52] Lentine KL Smith JM Hart A Miller J Skeans MA Larkin L OPTN/SRTR 2020 annual data report: kidney. Am J Transplant. (2022) 22(S2):21–136. (Special Issue: OPTN/SRTR Annual Data Report 2020). 10.1111/ajt.16982 35266618

[53] Ministry of Health, Labour and Welfare. “Zouki ishoku no jishijoukyou tou ni kansuru houkokusho (Report of Implementation Situations of Organ Transplantations) in Japanese (2021).

[54] Hanafusa N Abe M Joki N Hoshino J Kikuchi K Goto S 2020 annual dialysis data report, JSDT renal data registry. J Japan Soc Dial Ther. (2021) 54:611–57. in Japanese with English abstract. 10.4009/jsdt.54.611

[55] Nawata K Kimura M. Evaluation of medical costs of kidney diseases and risk factors in Japan. Health. (2017) 9:1734–49. 10.4236/health.2017.913127

[56] JMDC. “JMDC claims database.” (2022). Available at: https://www.jmdc.co.jp/en/jmdc-claims-database/

[57] Nawata K. An analysis of risk factors affecting cerebrovascular disease. Health. (2022) 14:866–82. (Erratum: Health, 14, 1038–1043.). 10.4236/health.2022.148061

[58] American Kidney Fund. “Symptoms, tests & treatments.” (2022). Available at: https://www.kidneyfund.org/all-about-kidneys/other-kidney-problems/protein-urine

[59] Mahfoud F Kandzari DE Kario K Townsend RR Weber MA Schmieder RE Long-term efficacy and safety of renal denervation in the presence of antihypertensive drugs (SPYRAL HTN-ON MED): a randomised, sham-controlled trial. Lancet. (2022) 399:1401–10. 10.1016/S0140-6736(22)00455-X 35390320

[60] Loh YC Chan SY Tew WY Oo CW Yam MF. New favonoid-based compound synthesis strategy for antihypertensive drug development. Life Sci. (2020) 249:117512. 10.1016/j.lfs.2020.117512 32145305

[61] Jackson RE Bellamy MC. Antihypertensive drugs. BJA Educ. (2015) 15:280–5. 10.1093/bjaceaccp/mku061

[62] Laurent S. Antihypertensive Drugs. Pharmacol Res. (2017) 124:116–25. 10.1016/j.phrs.2017.07.026 28780421

[63] Nawata K Ii M Kasai R. Over- and under-provision of diabetes screening: making more efficient use of healthcare resources. Public Policy Review. (2023) 19:1–36. Policy Research Institute, Ministry of Finance, Japan. 10.57520/prippr.19.1-1

[64] Fountain JH Kaur J Lappin SL. Physiology, Renin Angiotensin System. Treasure Island, FL: StatPearls [Internet] (2023). StatPearls Publishing. 29261862

[65] Lange-Jacobs PD Asma Shaikh-Kader A Thomas B Nyakudya T. An overview of the potential use of ethno-medicinal plants targeting the renin–angiotensin system in the treatment of hypertension. Molecules. (2020) 25:2114. 10.3390/molecules25092114 32366012 PMC7249071

[66] Ciftciler R Haznedaroglu IC. Pathobiological interactions of local bone marrow renin-angiotensin system and central nervous systemin systemic arterial hypertension. Front Endocrinol. (2020) 7:425. 10.3389/fendo.2020.00425 PMC7438890 32903745

[67] Lithell HOL. Effect of antihypertensive drugs on insulin, glucose, and lipid metabolism. Diabetes Care. (1991) 14:203–9. 10.2337/diacare.14.3.203 1828417

[68] Santos PCJL Krieger JE Pereira AC. Renin-angiotensin system, hypertension, and chronic kidney diseases: pharmacogenetic implications. J Pharmacol Sci. (2012) 120:77–88. 10.1254/jphs.12R03CR 23079502

[69] Loga-Zec S Asceric M Loga-Andrijic N Kapetanovic B Zerem E. The incidence of antihypertensive drug induced side effects in patients with diabetes mellitus type 2 and hypertension. Med Arch. (2014) 68:372–5. 10.5455/medarh.2014.68.372-375 25648509 PMC4314169

[70] Tedla YG Bautista LE. Drug side effect symptoms and adherence to antihypertensive medication. Am J Hypertens. (2016) 29:772–9. 10.1093/ajh/hpv185 26643686 PMC5863783

[71] Odigboegwu O Pan LJ Chatterjee P. Use of antihypertensive drugs during preeclampsia. Front Cardiovasc Med. (2018) 5:50. 10.3389/fcvm.2018.00050 29896480 PMC5987086

[72] Gill D Georgakis MK Koskeridis F Jiang L Feng Q Wei W Use of genetic variants related to antihypertensive drugs to inform on efficacy and side effects. Circulation. (2019) 140:270–9. 10.1161/CIRCULATIONAHA.118.038814 31234639 PMC6687408

[73] Suchard MA Schuemie MJ Krumholz HM You SC Chen R Pratt N Comprehensive comparative effectiveness and safety of first-line antihypertensive drug classes: a systematic, multinational, large-scale analysis. Lancet. (2019) 394:1816–26. 10.1016/S0140-6736(19)32317-7 31668726 PMC6924620

[74] Reeve E Jordan V Thompson W Sawan M Todd A Gammie TM Withdrawal of antihypertensive drugs in older people. Cochrane Database Syst Rev. (2020) 6:CD012572. 10.1002/14651858.CD012572.pub2 32519776 PMC7387859

[75] Jiang Q Chen Q Zhang T Liu M Duan S Sun X. The antihypertensive effects and potential molecular mechanism of microalgal angiotensin I-converting enzyme inhibitor-like peptides: a mini review. Int J Mol Sci. (2021) 22:4068. 10.3390/ijms22084068 33920763 PMC8071128

[76] Hassan D Peeters LEJ Koch BCP Versmissen J. Diffeences in antihypertenSive drug blood levels in patients with hypertensiON (DECISION): protocol for a prospective observational study comparing pharmacokinetics and pharmacodynamics between young and elderly patients. High Blood Press Cardiovasc Prev. (2022) 29:239–43. 10.1007/s40292-022-00505-w 35175576 PMC9050759

[77] CDC. “Diabetes and chronic kidney disease.” (2021). Available at: https://www.cdc.gov/diabetes/managing/diabetes-kidney-disease.html

[78] International Diabetes Foundation. “Diabetes complications.” (2022). Available at: https://www.idf.org/aboutdiabetes/complications.html

[79] Marcum ZA Fried LF. Aging and antihypertensive medication-related complications in the chronic kidney disease patient. Curr Opin Nephrol Hypertens. (2011) 20:449–56. 10.1097/MNH.0b013e32834902ad 21670671 PMC3531992

[80] Ptinopoulou AG Pikilidou MI Lasaridis AN. The effect of antihypertensive drugs on chronic kidney disease: a comprehensive review. Hypertens Res. (2013) 36:91–101. 10.1038/hr.2012.157 23051659

